# Timeframe for return to driving for patients with minimally invasive knee arthroplasty is associated with knee performance on functional tests

**DOI:** 10.1186/1471-2474-15-198

**Published:** 2014-06-10

**Authors:** Hsuan-Ti Huang, Jing-Min Liang, Wei-Tso Hung, Ying-Yi Chen, Lan-Yuen Guo, Wen-Lan Wu

**Affiliations:** 1 Division of Adult Reconstruction Surgery, Department of Orthopaedics, Kaohsiung Medical University Hospital, Kaohsiung, Taiwan; 2 Department of Sports Medicine, College of Medicine, Kaohsiung Medical University, Kaohsiung 701, Taiwan

**Keywords:** Knee Osteoarthritis, Total knee arthroplasty, Braking reaction time, Knee functional test

## Abstract

**Background:**

This study hopes to establish the timeframe for a safe return to driving under different speed conditions for patients after minimally invasive total knee arthroplasty and further explores how well various kinds of functional tests on knee performance can predict the patients’ braking ability.

**Methods:**

14 patients with right knee osteoarthritis were included in the present study and instructed to perform three simulated driving tasks at preoperative, 2 weeks postoperative and 4 weeks postoperative.

**Results:**

The results showed that the total braking time at 4 week postoperative has attained the preoperative level at the driving speed 50 and 70 km/hr but not at the driving speed 90 km/hr. It had significantly improving in knee reaction time and maximum isometric force at 4 weeks postoperative. Besides, there was a moderate to high correlation between the scores of the step counts and the total braking time.

**Conclusions:**

Summary, it is recommended that driving may be resumed 4 weeks after a right knee replacement but had to drive at low or moderate speed and the best predictor of safety driving is step counts.

## Background

Total knee arthroplasty (total knee replacement, TKA) has proved to be a successful treatment for patients suffering knee osteoarthritis. TKA can effectively improve physical functional performance [1-3], reduce pain and improve quality of life [4-6]. However, this procedure traditionally required an extensive approach (20–25 cm) with an arduous recovery period and it could cause the weakness of the knee extensors, impaired proprioception, and negatively affect functional performance [7,8]. Traditional TKA might cause to the prolonged rehabilitation phase and had less functional outcome. Minimally invasive surgery total knee arthroplasty (MIS-TKA) is a new surgical technique which involves the use of a smaller incision than the one used in traditional knee replacement. The benefits of this procedure includes: improved gain of knee joint range of motion, improved quadriceps function, shortened length of hospital stay, early recovery and rapid rehabilitation [7,8]. In past study, patients with MIS-TKA regain the ability of complex function like walking without assistance at 32 days postoperatively. However, it had to take 45 days in the traditional TKA group [9].

Following orthopaedic surgery (e.g. total knee arthroplasty), patients are “temporarily” unable to drive [10]. After hospital discharge, patients are often eager to return to driving, which allows them to resume their social and recreational activities or even to return to work. According to past study, there were less evidences and standards to assess the patients following MIS-TKA when they want solo driving. In order to drive safely, the most important of all is moving lower limbs as smooth and quick as possible to overcome an emergency situation. In order to move lower limb smooth, drivers must have enough muscle strength and good knee joint proprioception, but these abilities will decrease following TKA. According to past study, brake reaction time is often divided into gas-off time, transition time, and total reaction time [11]. Gas-off time counts from the time of information processing, decision-making, to the time of muscle activation to release the accelerator pedal. Transition time was the time of lower limb moved from the accelerator pedal to the brake pedal. In the past study, it concluded that there were many factors could affect the brake reaction time, such as age [12], gender [13], visual ability [14,15], task complexity [16,17], muscle force [18], and drug intake [19].

For discussing the timeframe for return to driving for patients with traditional knee arthroplasty, the Spalding et al. [20] found that the braking reaction time returned at the time of 8 weeks after surgery for the right knee. Therefore, the authors suggested that driving may be resumed 8 weeks after a right knee replacement. Pierson et al. [21] had even reported that braking reaction time showed significantly faster than preoperative values at 6 weeks postoperative. Therefore, the authors permit driving at 6 weeks postoperatively for all TKAs. Besides, there was also study reported that braking reaction time was poorer at 10 days after surgery, but returned to preoperative values after right knee surgery at 30 days postoperative [17]. In particular, Liebensteiner et al.’s recommendation [22] of relaxing driving abstinence after two weeks postoperatively for patients with right-sided TKA is in contrast to previously expressed conclusions. A potential reason for the inconsistency with previous studies was that they only considered the proportion of patients above a danger threshold. In fact, their patients still showed a significant increase in braking reaction time from pre-operative to 2 weeks postoperative, meaning that patients were not fully recovered at this stage. In a recent study of people who had undergone MIS-TKA, Dalury et al. [11] reported that surgeons might consider allowing patients who was treated with contemporary right TKAs using a limited extensor mechanism approach to drive 4 weeks after surgery. To our knowledge, this study is to date the only one which has explored the braking reaction time after MIS-TKA. However, the driving task in their study only arranged at a low driving speed of 30 miles/hr (approximately 48 km/hr). In addition, the results of functional tests to quantify recovery are important in assessing the driving function. Hence, this study investigates the impact of driving speeds on the timeframe for a safe return to driving for patients after minimally invasive total knee arthroplasty and further explores how well various kinds of functional tests on knee performance can predict the patients’ braking ability.

## Methods

### Subject

A one-group pretest-posttest design was used in this study to determine the timeframe for a safe return to driving under different speed conditions for patients after receiving minimally invasive total knee arthroplasty. 14 severe right knee osteoarthritis patients (4 male and 10 female, mean age was 63.14 ± 6.62) without any neuromuscular disorder, cardiovascular disease and had normal visual field were recruited in this study. Each participant had a driving licence and had driven a motor vehicle for more than 5 years. All patients were able to drive preoperatively. These participants were diagnosed as Knee OA by an orthopaedic surgeon in Kaohsiung Medical Hospital and will accept MIS-TKA within three months. Their radiographic finding of the knee according to the Kellgren & Lawrence classification method was grade 3 or more and there were no outliers in the mechanical axis (deviation > 3°) in any of the patients, therefore, they did not require a lateral release. All of the patients in this study then received the same treatment by MIS-TKA technique using the Zimmer LPS-flex posterior stabilized primary total knee prosthesis (Zimmer, Inc., Warsaw, Poland). A midline skin incision was made that ranged from 9–12 cm in length. The arthrotomy was done through a mini-parapatella approach that extended the arthrotomy less than 3 cm into the quadriceps tendon. The average operative time was 74 minutes, and ranged from 56 minutes to 93 minutes. The tourniquet was removed immediately after incision closure. After the operation, all patients received the same pain control program with one shot of femoral nerve PCA and Cox-2 NSAID pain medication. After discharge, Rivaroxaban 10 mg for 14 days was given to all patients prophylactically to prevent venous thromboembolism and pulmonary embolism. On the whole, there were no cases of postoperative complications such as infection, thrombovascular events, instability, or fractures in these subjects. The inpatient rehabilitation program was the same for all patients including ambulation with walker and continuous passive motion exercise with machine. No patient attended an outpatient rehabilitation program after discharge. Patients only received a patient education sheet before discharge for subsequent reminders. All participants provided written informed consent prior to participation and all procedures were reviewed and approved by the Institutional Review Board of Kaohsiung Medical University Chung-Ho Memorial Hospital for the use of human subjects in research. Baseline measures were arranged within 2 weeks preoperative and follow-up at 2 and 4 weeks postoperative.

### Equipment

#### Driving simulator

The driving simulator (PITOTECH CO., LTD, Taiwan) is an assessment tool that one of the functions of this simulator is to evaluate the emergency brake response time during driving. This simulator consisting of actual brake and accelerator pedal assemblies which attached to an automobile steering column was constructed. A 42-in color monitor that displayed a driving situation was placed in front of the steering wheel. The monitor was positioned at the subject’s eye level and the steering wheel was placed at a height to represent his or her normal driving position. An automobile seat was adjustable for the patient’s height.

#### Neuromuscular testing equipment of the knee

In this study, a leg extension system (Leg Extension, Monitored Rehab Systems, Harlem in the Netherlands) was used to measure the proprioception, response time and maximum extension force for knee joint. This system was especially designed for optimal open chain reconditioning, rehabilitation, and testing of the knee joint. The eccentric cam provides optimal quadriceps loading in the total range of motion. Range limiters in both directions make it easy to train or test within a safe range of motion.

#### Clinical tests of the knee

For clinical tests of the knee function, a chair and a stopwatch were used for Timed Up and Go Test (TUG test) and a 15-cm step was used for Step test.

### Procedures

To investigate the relationship between the performance on a functional performance test and the driving performance for the people suffered knee OA, the present study measured the functional data from clinical TUG test and step test, and neuromuscular mechanical test of maximum isometric knee extension force, knee reaction time, knee proprioception by one clinical technician. In driving performance test, the emergency brake response (brake reaction time) was recorded. For each subject, alcohol intake is prohibited from 24 hours prior to the test day.

#### Driving task

The driving task is to drive a virtual car using the steering wheel, accelerator and brake pedals (automatic clutch) at a driving speed of 50, 70 and 90 km/hr. Subjects are required to increase the speed to each target speed and then keep the speed from ± 5 km/hr. While the target speed was achieved, a pedestrian suddenly appeared and trotted from the right side to the center of screen. At that instant, subjects were required to release the accelerator and press the brake pedal as soon as possible to avoid the crash. Five seconds after a deceleration, subjects were instructed to return target speed and wait the next pedestrian appeared. Totally, it had ten events for each driving speed condition.

#### Knee proprioception test

The knee proprioception tests were carried out by using the Monitored Rehab Systems - Leg Extension. Subjects were asked to sit on the chair and keep the knee angle at degree of 90. In each test, subjects had three times practices to be familiar with testing measure. In the knee proprioception test, firstly subjects were asked to do their best to do the maximum knee extension and the testing range was set at the 30% of maximum knee extension. In an initial experiment, subjects had to raise their non-involved leg to the target height with visual feedback and keep the position for 7 seconds and put down the leg to rest 5 seconds and repeated the same measure again. Afterwards, subjects were asked to do the same test measure without visual feedback. While the non-involved leg had been tested, the involved leg was tested by the same testing measure. Totally, subjects would perform two repetitions for each leg. In this test measure, the proprioception deficit was calculated by the position error of subject’s leg. Subjects had to raise their legs to the target height and keep the precise position without any sway.

#### Knee joint response test

The knee joint response test was also tested by using the Monitored Rehab Systems at the sitting position, subjects asked to control a cursor left by knee flexion and right by knee extension. At first, subjects had to move the cursor to the target zone and keep at the position for 1 second. Next, the target zone disappeared and appeared again at another position. Subjects were asked to quickly move the cursor to the target zone again and keep at the new position for 1 second. The testing was measured three times at one trail and the non-involved leg and involved leg were tested separately. In this study, the shortest complete times in each trial was recorded and defined as the response time (RT). Therefore, each subject was asked to do the testing as quickly as possible.

#### Maximum isometric knee extension force test

The maximum isometric knee extension force test was also tested by using the Monitored Rehab Systems at the sitting position, subjects had to do their best to do knee extension and keep the force for 4 seconds. Subjects had to perform this test measure twice and the non-involved leg and involved leg were tested separately. The maximum force during 4 seconds for each test measure was recorded.

#### Timed Up and Go Test (TUG test)

The TUG test is a simple test used to assess a person’s mobility and requires both static and dynamic balance. This function is also dependent on good lower-limb muscle strength and joint mobility. Firstly, this test was measured with the subject sitting correctly in a chair, the subject’s back should rest on the back of the chair and their arms rest on the arm rests. Subjects were asked to stand up, walk to the marker on the floor 3 meters away from the chair, turn around and walk back to the chair and sit down as quickly as possible [23]. The test was measured three times and recorded the complete times.

#### Step test

The step test, also called single-step test [24], is a reliable tool for evaluation of the unilateral limb functional ability following lower limb surgery [25]. Participants were instructed to maintain balance on involved leg, whilst stepping the contralateral limb as quickly as possible on and off a 15-cm step in front of the subject. The number of counts that subjects could place the foot up onto the step and return it to the floor over a 15 s interval was recorded. Each subject had to perform three times on this task. It was used to measure the ability of dynamic balance and the lower-limb muscle strength for patients with knee osteoarthritis and it showed significantly fewer steps when standing on the osteoarthritic limb for knee OA patients compared with controls [26].

### Data analysis

#### Brake reaction test

The onset of a pedestrian started crossing the street will be setting as the stimulus signal onset. The brake response time was divided into three parts: gas-off time, transition time, and pressing time. Gas-off time was calculated as the time difference between the stimulus onset and full leave of accelerator pedal. The transition time was calculated as the time difference between full leave of accelerator pedal and the first touch to the brake pedal. The pressing time was calculated as the time difference between the first touch to the brake pedal and full press of brake pedal. The total braking time was calculated as the time difference between the stimulus onset and the full press of brake pedal.

#### Neuromuscular testing of the knee

For functional neuromuscular mechanical test, the proprioception deficit (%) was calculated as follows:

$$\left(P_{\mathrm{nonv}}-P_{v}\right)/P_{v}\times 100\%$$

P_nonv_ → the average errors of position without visual feedback

P_v_ → the average errors of position with visual feedback

The knee joint response test recorded the fastest response times (RT, s) from signal appear to the time the subject placed the cursor to the target zone. Maximum force (N) measured in maximum isometric knee extension test was calculated as the average of peak force (N) of two tests for each leg.

For clinical test of the knee, the TUG test recorded the complete time (s) of each test and calculated the average time for all three tests. The step test recorded the step counts for 15 seconds and finally the data that go into analysis was the highest individual score in the three trials.

### Statistical analyses

SPSS statistical software (SPSS Inc., USA) was used in this study for statistical analysis. The Wilcoxon signed-rank test was used to compare the differences in outcome measures of preoperative and postoperative results, and the differences between two postoperative progresses. The pre-operative function scores on the operative side were compared to the non-operative side with the paired sample Wilcoxon signed rank test. The Spearman’s rho was used to verify the correlation between the results of knee joint functional tests and brake response time test for all stages. It was also used to verify the correlation between the results of each knee joint functional tests. A statistical significance level was set at p < 0.05.

## Result

The result of brake reaction time in difference driving speed was showed in Table 1. Although there was not found significant difference in total braking time between each of three phases in any driving speed condition, we found out that the total braking time has attained the preoperative level at the driving speed 50 and 70 km/hr but not at the driving speed 90 km/hr. The results showed that the total braking time is still slower than the preoperated braking time in the driving speed of 90 km/hr (2.12 sec vs. 1.92 sec). In addition, for 50 km/hr condition, the transition phase showed significantly faster response time in 4 weeks than 2 weeks postoperative. For 70 km/hr condition, there were no significant differences in three phases. For 90 km/hr condition, the transition phase showed significantly faster response time in preoperative than 2 weeks postoperative. In addition, the pressing response in 4 weeks postoperative showed significantly slower than preoperative.

**Table 1:** The results of brake reaction times at preoperative and postoperative during three driving speed

		**Preoperative**	**2 weeks**	**4 weeks**
50 km/hr	Gas-off time (s)	1.33(0.99/1.48)	1.36(0.70/1.66)	1.28(0.76/1.39)
	Transition time (s)	0.30(0.15/0.64)	0.36(0.19/0.71)	0.29(0.19/0.49)*
	Pressing time (s)	0.23(0.12/0.44)	0.26(0.16/0.44)	0.22(0.15/0.33)
	Total braking time (s)	1.93(1.26/2.52)	2.28(1.04/2.66)	1.86(1.10/2.19)
70 km/hr	Gas-off time (s)	1.26(0.85/1.58)	1.10(0.81/1.67)	0.93(0.88/1.66)
	Transition time (s)	0.16(0.14/0.42)	0.29(0.15/0.40)	0.23(0.14/0.40)
	Pressing time (s)	0.19(0.08/0.48)	0.26(0.15/0.35)	0.20(0.14/0.37)
	Total braking time (s)	2.02(1.11/2.23)	1.89(1.22/2.19)	1.77(1.21/2.18)
90 km/hr	Gas-off time (s)	1.50(1.33/1.60)	1.62(1.03/1.75)	1.28(0.76/1.39)
	Transition time (s)	0.17(0.13/0.36)	0.29(0.19/0.46)†	0.28(0.18/0.38)
	Pressing time (s)	0.11(0.08/0.38)	0.19(0.17/0.32)	0.26(0.15/0.45)†
	Total braking time (s)	1.92(1.57/2.25)	2.02(1.48/2.48)	2.12(1.43/2.27)

†means the significant difference that compared to preoperative.

*means the significant difference that compared to 2 weeks postoperative.

The results of proprioception deficit, response time, maximum isometric force for operative leg and non-operative leg, and physical activity measure of TUG test and step counts were showed in Table 2. The non-operative side showed significantly better results in the following tests: maximum isometric knee extension force test and knee joint response test, but the result did not reveal any significant differences in knee proprioception test. For operative leg, the maximum isometric force for knee extension in preoperative showed significantly greater than 2 weeks and 4 weeks postoperative. It also showed significant difference between 2 weeks and 4 weeks postoperative. For knee joint response test, the result of response times showed significantly faster in 4 weeks than 2 weeks postoperative. For proprioception, TUG, and step count tests, they did not show any significant difference in three phases.

**Table 2:** The results of knee functional scores at preoperative and postoperative for operative and non-operative side

		**Preoperative**	**2 weeks**	**4 weeks**
Operative side	Proprioception deficit (%)	29.63(13.28/45.67)	44.44(30.13/53.82)	30.00(15.09/37.53)
	Maximum force (N)	447(310.5/593.5)‡	171(114/217)†	272(213/315)†*
	RT (s)	0.91(0.78/1.17)‡	1.02(0.80/1.41)	0.87(0.75/1.06)*
Non-operative side	Proprioception deficit (%)	23.08(12.92/44.14)	27.72(21.19/37.99)	26.92(15.16/40.68)
	Maximum force (N)	740(643.25/862.5)	725(680/805.5)	746(674/886)
	RT (s)	0.79(0.72/0.87)	0.83(0.67/0.88)	0.80(0.75/0.91)
	TUG test (s)	11.69(10.72/14.41)	13.00(10.47/16.27)	11.90(10.12/13.30)
	Step counts	9.00(9.00/11.50)	9.00(9.00/10.50)	10.00(9.00/12.00)

‡means the significant difference that compared to non-operative side.

†means the significant difference that compared to preoperative.

*means the significant difference that compared to 2 weeks postoperative.

The correlations between the results of knee joint functional tests and brake reaction test for all stages were showed in Table 3. For both 50 and 70 km/hr conditions, the pressing time and total braking time showed significant correlation with step counts and the pressing time additionally showed significant correlation with proprioception deficit. In addition, the transition time showed significant correlation with response time in knee joint response test in 70 km/hr condition. For 90 km/hr condition, the gas-off time, transition time, and total braking time all showed significant correlation with step counts. The correlations between the results of each knee joint functional tests were showed in Table 4. Maximum Force was significantly correlated with the scores of step counts and TUG Test.

**Table 3:** The correlation of function test and braking reaction while driving at three speed conditions

	**Gas-off time**	**Transition time**	**Pressing time**	**Total braking time**
*50 km/hr*				
Proprioception deficit	.145	.352	.520*	.313
Maximum force	-.059	-.299	-.154	-.173
RT	.220	.434	.371	.353
Step counts	-.507	-.418	-.510	-.532*
TUG test	-.201	.316	.098	.028
*70 km/hr*				
Proprioception deficit	.058	.394	.554*	.336
Maximum force	-.099	-.293	-.032	-.166
RT	.015	.521*	.160	.195
Step counts	-.503	-.422	-.423	-.659**
TUG test	-.340	.253	-.068	-.233
*90 km/hr*				
Proprioception deficit	.307	.484	.349	.459
Maximum force	.034	-.377	-.180	-.134
RT	.217	.507	.177	.352
Step counts	-.625*	-.566*	-.405	-.732**
TUG test	-.219	.080	-.048	-.155

*means the *p* value < 0.05.

**means the *p* value < 0.01.

**Table 4:** The correlation between the results of each knee joint functional tests

	**TUG test**	**Step counts**	**RT**	**Maximum force**
Proprioception deficit	.262	-.187	.011	-.235
Maximum force	-.503**	.442*	-.270	-
RT	.371	-.353	-	-
Step counts	-.261	-	-	-

*means the *p* value < 0.05.

**means the *p* value < 0.01.

## Discussion

In the present study, the total braking time was not found significant difference between each of three phases in any driving speed condition. But, the results showed that the total braking time is still slower than the preoperated reaction time in the driving speed of 90 km/hr (2.12 sec vs. 1.92 sec). In the driving speed of 50 km/hr, the transition time showed significantly improved that compared to 2 weeks postoperative at 4 weeks postoperative (0.36 vs. 0.29 s). Besides, in the moderate speed (70 km/hr) driving condition, there were not found any significant difference between each of the three phases. However, in the high speed driving condition (90 km/hr), the transition time showed significantly slower than preoperative at 2 weeks postoperative (0.17 vs. 0.29 s) and the time also didn’t recover back to preoperative level at 4 week postoperative (0.17 vs. 0.28 s), even though there were no significant differences. The pressing time also showed significantly slower than preoperative at 4 weeks postoperative (0.11 vs. 0.26 s). These results showed that in the low or moderate speed driving condition, patients suffering TKA would have better performance than high speed driving condition. Therefore, in the present study, we suggested that driving may be resumed 4 weeks after a right knee minimally invasive arthroplasty but had to drive at low or moderate speed, such as driving at the urban areas or suburbs. The result was consistent with that reported by Dalury et.al in 2011. They concluded that when people underwent contemporary right TKAs (defined as limited soft tissue disruption, multimodal pain management protocols, and intense postoperative rehabilitation), there was a quicker recovery to baseline compared with more traditional TKA [16,17,20,21] and a return to safe driving was suggested at about 4 weeks postoperatively [11]. However, Dalury’s study only measured the brake response times at a driving speed of 30 miles per hour. Our study, focusing on the similar MIS-TKA, shows that high-speed driving earlier than 4 weeks is not recommended, in order to avoid accidents. Driving on the highway is not recommended at 4 weeks postoperative. On the other hand, Dalury also reported that few patients passed an additional driving test at 2 or 3 weeks postoperative [11]. In the present study, we also found that three patients’ total braking time was faster than preoperative at 2 weeks postoperative under low and moderate speed conditions. This result would suggest that some patients who underwent right MIS-TKA may drive well at 2 weeks postoperative, but more rigorous assessment is needed to address safety concerns. Therefore, we still recommend that right MIS-TKA patients do not drive until 4 weeks postoperative, and then only under low and moderate speed conditions.

According to the correlation between the results of knee functional scores and driving braking reaction time, we could find that the transition time showed a positive correlation with knee reaction time during moderate driving speed conditions. In this present study, the knee reaction time test was designed to measure the moving ability for knee joint by controlling a cursor to the target zone as quick as possible. Therefore, the movement was more similar to the braking movement of transition that was the time period from the full leave of accelerator pedal to the first touch to the brake pedal. It requires the contributions of the quick motions of the affected knee. On the other hand, proprioception deficit had a moderate positive correlation with pressing time during low (r = 0.520) and moderate (r = 0.554) speed conditions. It reflected that higher proprioception deficit would cause a longer pressing time. However, with the increasing of test difficulty, proprioceptive deficit when compared to other functional loss is mildly affected. Therefore, the pressing time didn’t showed significant correlation with proprioception deficit in 90 km/hr condition. On the other hand, the result of step-test which was an integrated functional test showed a moderate to high negative correlation with total braking time in low (r = -0.532), moderate (r = -0.659), and high (r = -0.732) speed conditions and with gas-off time (r = -0.625) and transition time (r = -0.566) only in high speed condition. It represented that better lower limb function of their operated limb assessed with single-step test could make more excellent test result in each test condition and finally displayed a quick total braking response. Instead, TUG score and maximal force alone are not good predictors of braking function. Firstly, we know that the score of TUG test are associated with the muscle strength of both legs. This test cannot discriminate the separated contribution of involved side and non-involved side. Instead of that, step test can be used to measure the dynamic balance of single limb stance. But it is difficult to say that the step tests evaluate only balance for the subjects, since the results also relate to lower-limb muscle strength and ability. This study also has verified that lower-extremity muscle force was significantly correlated with the scores of step counts and TUG Test (Table 4). Therefore, step test score can be indirectly reflect the level of the lower-limb muscle strength. Insufficient muscle strength and endurance will lead to an inability to support the weight of their body and cause the poor performance on this test. In this study, the maximum isometric force at 4 weeks postoperative showed significant improving than 2 weeks postoperative but it still did not recover to the preoperative level. Failure of volitional activation of quadriceps femoris muscle may play an important role in the cause of the decreased force production in patients following TKA [27,28]. Therefore, although the result of this study seems to indicate that maximal muscle force is not required to execute braking movement, insufficient muscle strength may also increase driving crash risk.

A limitation of this study is that we didn’t actively recruit volunteers with the same background (age, BMI, gender, preoperative results, etc.). These confounding factors may well affect the results of this study and influence the application or interpretation of the results. In addition, the validity of our study is limited due to a small sample size and narrow age range (from 54 to 75 years old). Caution must also be applied, as the findings might not be transferable to MIS-TKA patients younger than our age range. However, it has been reported that there has recently been a sharp increase in the number of total knee arthroplasty (TKA) procedures performed in younger patients [29]. Therefore, we should also address this problem. With regard to the small sample size, a post hoc power analysis revealed that the statistical power of this study reached a power level of 0.8 for data showing significant differences. This means our sample size is enough to warrant these assumptions. Conversely, non-significant results in our sample are very likely related to the limited statistical power of the analysis. More data from future trials will make it possible to promote statistically significant findings. Furthermore, all patients in this study only performed basic rehabilitation exercises such as ambulation with walker and continuous passive motion exercise with machine from the first postoperative day until the fifth postoperative day (discharged). Hence, caution must be applied, as the findings may not be transferable to MIS-TKA patients who took part in rehabilitation with an enhanced recovery program. Additionally, from our experimental observations and previous survey studies [22,30], it has been found that leg kinematics might also be a confounder influencing braking time. It is possible to complete the process of braking by flexing the right leg at all major joints while lifting the entire foot from the floor, then adducting the leg, but it is also possible to brake by lifting just the right forefoot by dorsiflexion. It was noted that the fastest BRT was in subjects who solved the task by only moving the foot [30]. Therefore, monitoring kinematics may yield interesting data.

## Conclusions

Summary, according to the results of the present study, 4 weeks after knee minimally invasive arthroplasty showed significant improvement of knee function as reflected in reaction time and maximum force, however, the maximum isometric force for knee extension did not achieve the preoperative level. Best predictor of safety driving is step counts. Besides, the braking reaction time was also showed similar results to the knee functional test. Therefore, if patients following TKA requirements for driving, surgeons may consider allowing patients treated with minimally invasive right TKAs to drive 4 weeks after surgery under low or moderate speed conditions. High-speed driving earlier than 4 weeks is not recommended.

## Competing interests

The authors declare that they have no competing interests.

## Authors’ contributions

HTH and WLW participated in the research conception and planned the study design. JML, WTH, and YYC performed the literature search and extracted relevant data. HTH, WLW, and LYG participated in the statistical analyses and interpretation of result, and in the preparation and revision of this manuscript. All authors were involved revisions to the manuscript and final approval of the version to be published.

## Pre-publication history

The pre-publication history for this paper can be accessed here:

http://www.biomedcentral.com/1471-2474/15/198/prepub
